# Draft Genome Sequence of NYR20, a Red Pigment-Secreting Mutant of Saccharomyces cerevisiae

**DOI:** 10.1128/MRA.01161-20

**Published:** 2021-01-07

**Authors:** Hiro Takahashi, Shin-ichi Iwaguchi, Hisashi Kondo, Taichiro Motomura, Masataka Murase, Anna Takahashi, Shuichi Fukuyoshi, Chiyoko Machida, Shin Kanamasa, Satoru Yamamoto, Takayuki Yoshizaki

**Affiliations:** a Graduate School of Medical Sciences, Kanazawa University, Kanazawa, Ishikawa, Japan; b Graduate School of Horticulture, Chiba University, Matsudo, Chiba, Japan; c Fundamental Innovative Oncology Core Center, National Cancer Center, Chuo-ku, Tokyo, Japan; d Faculty of Science, Nara Women’s University, Nara, Nara, Japan; e Faculty of Life Science and Biotechnology, Fukuyama University, Fukuyama, Hiroshima, Japan; f Faculty of Information Technologies and Control, Belarusian State University of Informatics and Radio Electronics, Minsk, Belarus; g College of Bioscience and Biotechnology, Chubu University, Kasugai, Aichi, Japan; h Institute of Medical, Pharmaceutical and Health Sciences, Kanazawa University, Kanazawa, Ishikawa, Japan; i Graduate School of Bioscience and Biotechnology, Chubu University, Kasugai, Aichi, Japan; Vanderbilt University

## Abstract

A Saccharomyces cerevisiae mutant strain, NYR20, produces a red pigment owing to adenine auxotrophy. Unlike other yeast adenine biosynthetic mutants, this strain not only produces but also secretes this pigment. Here, we report the NYR20 draft genome sequence, thereby advancing our understanding of pigment secretion mechanisms.

## ANNOUNCEMENT

Adenine deficiency results in the accumulation of polyribosylaminoimidazole, a red pigment, in the vacuole of yeast adenine biosynthetic mutants *ade1* and *ade2* (1). A mutant strain of P-684 (a Saccharomyces cerevisiae strain isolated from the flowers of Prunus verecunda ‘Antiqua’ [2]), which produces and secretes a red pigment, was obtained and designated NYR20 (3). For this, we sporulated P-684 and obtained high ethanol-producing strains, which were then treated with ethyl methanesulfonate. Red colonies (exhibiting the adenine auxotrophic phenotype) were selected, and NYR20, a pigment-secreting strain, was isolated from the colonies. The Japanese refined sake prepared with NYR20 is red in color (3). Thus, to investigate the underlying mechanisms governing pigment secretion, we analyzed the genome sequence of NYR20.

NYR20 cells were cultured in YPAD medium (1% yeast extract, 2% Bacto peptone, 2% glucose, 0.04% adenine sulfate) at 25°C. After collection, their genomic DNA was isolated using a Dr. GenTLE (from yeast) high-recovery kit (TaKaRa Bio, Inc.). A DNA library was prepared using a DNA library preparation kit (Beijing Genomics Institute, Shenzhen, China). DNA fragmentation was performed by using a g-TUBE (Covaris, Inc.) to produce fragments having an average length of 300 bp, followed by end repair, A tailing, adaptor ligation, and PCR; DNA library purification was performed according to the manufacturer’s instructions. Paired-end sequencing on the DNBSEQ-G400 platform (MGI Tech, Shenzhen, China) generated 16,720,478 reads (150-bp reads). Adaptor sequences were removed and low-quality reads were trimmed from the obtained short reads using fastp version 0.19.10 (4) with default parameters. *De novo* sequence assembly was performed using SPAdes version 3.14.0 (5) with the “merged” and “isolate” parameters enabled. A reference-guided scaffolding of the draft genome sequence was conducted using RaGOO version 1.1 (6), with default parameters and the S. cerevisiae S288c genome sequence (GenBank accession number GCF_000146045.2) as the reference genome. Among the RaGOO-generated scaffolds, a concatenated, nonlocalized scaffold was discarded, as it consisted of short, highly fragmented contigs with no homology to the reference sequence. The resulting genome assembly was 11,518,743 bp long and was divided into 17 scaffolds comprising 220 contigs. The *N*_50_ values (contig and scaffold), GC content, and genome coverage were 114,682 bp and 892,137 bp, 38.1%, and 208.3×, respectively. Of the 1,711 benchmarking universal single-copy ortholog (BUSCO) genes, 98.3% (including 0.6% duplicated genes) were found in the assembly, as calculated by BUSCO version 3.1.0 (7), using the “sp=saccharomyces_cerevisiae_S288C” parameter and the “saccharomycetales_odb9” data set. Coding region prediction for the scaffolds was conducted using AUGUSTUS version 3.3.3 (8) with the parameters “noInFrameStop=true,” “genemodel=complete,” and “species=saccharomyces_cerevisiae_S288C” enabled. The estimated number of genes in the draft genome was 5,324. Of these, 64 genes with incomplete stop codons were discarded in the data registration step in the database. Gene annotation was also performed using Trinotate version 3.2.1 (9) with default parameters.

These data can provide insights into the genetic basis of the red coloration observed when NYR20 is used for brewing.

### Data availability.

The draft genome sequence and gene annotation for NYR20 are deposited in GenBank/ENA/DDBJ under accession number BLZP00000000 (BLZP01000001 through BLZP01000017). The SRA/DRA/ERA accession number is DRA010486.
